# Effects of ventilation strategy on distribution of lung inflammatory cell activity

**DOI:** 10.1186/cc12854

**Published:** 2013-08-15

**Authors:** Nicolas de Prost, Eduardo L Costa, Tyler Wellman, Guido Musch, Mauro R Tucci, Tilo Winkler, R Scott Harris, Jose G Venegas, Brian P Kavanagh, Marcos F Vidal Melo

**Affiliations:** 1 Department of Anesthesia, Critical Care and Pain Medicine, Massachusetts General Hospital, Harvard Medical School, 55 Fruit Street, Boston, MA 02114, USA; 2 Department of Medicine (Pulmonary and Critical Care Unit), Massachusetts General Hospital, Harvard Medical School, 55 Fruit Street, Boston, MA 02114, USA; 3 Departments of Critical Care and Anesthesia, Hospital for Sick Children, University of Toronto, 555 University Avenue, Toronto, ON, M5G 1X8, Canada

## Abstract

**Introduction:**

Leukocyte infiltration is central to the development of acute lung injury, but it is not known how mechanical ventilation strategy alters the distribution or activation of inflammatory cells. We explored how protective (vs. injurious) ventilation alters the magnitude and distribution of lung leukocyte activation following systemic endotoxin administration.

**Methods:**

Anesthetized sheep received intravenous endotoxin (10 ng/kg/min) followed by 2 h of either injurious or protective mechanical ventilation (*n *= 6 per group). We used positron emission tomography to obtain images of regional perfusion and shunting with infused ^13^N[nitrogen]-saline and images of neutrophilic inflammation with ^18^F-fluorodeoxyglucose (^18^F-FDG). The Sokoloff model was used to quantify ^18^F-FDG uptake (*K*_i_), as well as its components: the phosphorylation rate (*k*_3_, a surrogate of hexokinase activity) and the distribution volume of ^18^F-FDG (*F*_e_) as a fraction of lung volume (*K*_i _= *F*_e _× *k*_3_). Regional gas fractions (*f*_gas_) were assessed by examining transmission scans.

**Results:**

Before endotoxin administration, protective (vs. injurious) ventilation was associated with a higher ratio of partial pressure of oxygen in arterial blood to fraction of inspired oxygen (PaO_2_/FiO_2_) (351 ± 117 vs. 255 ± 74 mmHg; *P *< 0.01) and higher whole-lung *f*_gas _(0.71 ± 0.12 vs. 0.48 ± 0.08; *P *= 0.004), as well as, in dependent regions, lower shunt fractions. Following 2 h of endotoxemia, PaO_2_/FiO_2 _ratios decreased in both groups, but more so with injurious ventilation, which also increased the shunt fraction in dependent lung. Protective ventilation resulted in less nonaerated lung (20-fold; *P *< 0.01) and more normally aerated lung (14-fold; *P *< 0.01). *K*_i _was lower during protective (vs. injurious) ventilation, especially in dependent lung regions (0.0075 ± 0.0043/min vs. 0.0157 ± 0.0072/min; *P *< 0.01). ^18^F-FDG phosphorylation rate (*k*_3_) was twofold higher with injurious ventilation and accounted for most of the between-group difference in *K*_i_. Dependent regions of the protective ventilation group exhibited lower *k*_3 _values per neutrophil than those in the injurious ventilation group (*P *= 0.01). In contrast, *F*_e _was not affected by ventilation strategy (*P *= 0.52). Lung neutrophil counts were not different between groups, even when regional inflation was accounted for.

**Conclusions:**

During systemic endotoxemia, protective ventilation may reduce the magnitude and heterogeneity of pulmonary inflammatory cell metabolic activity in early lung injury and may improve gas exchange through its effects predominantly in dependent lung regions. Such effects are likely related to a reduction in the metabolic activity, but not in the number, of lung-infiltrating neutrophils.

## Introduction

There is little information on the effects of different mechanical ventilation strategies on *in vivo *regional lung inflammation. Nonetheless, reduction in regional inflammation is frequently proposed as the rationale for the benefit associated with protective ventilation in patients [1-3].

Pulmonary neutrophilic inflammation, a major process in the early stages of acute lung injury (ALI) [4,5], is increasingly being assessed by measuring the net ^18^F-fluorodeoxyglucose (^18^F-FDG) uptake rate (*K*_i_) using positron emission tomography (PET) [6-11]. The current concept during ALI, derived from experimental studies, is that ^18^F-FDG uptake is determined predominantly by the combination of the absolute number of lung-infiltrating neutrophils and their metabolic activity [7,10,12]. In terms of kinetics modeling, *K*_i _is the product of two parameters: the phosphorylation rate constant (*k*_3_, a surrogate of hexokinase activity) and the distribution volume of ^18^F-FDG as a fraction of lung volume (*F*_e_) [13]. Accordingly, a similar net ^18^F-FDG uptake rate could result from a large number of inflammatory cells with low metabolic activity (low *k*_3_) as well as from a smaller number of cells with high metabolic activity (high *k*_3_). Such distinct conditions could represent different pathophysiological mechanisms through which mechanical ventilation strategies alter lung neutrophilic inflammation. Indeed, different biological features in the presence of distinct *k*_3 _have been observed in fields where ^18^F-FDG kinetics has previously been used, such as oncology. For instance, a high value of *k*_3 _was associated with poor prognosis in leukocyte malignancies [14,15], and a low value of *k*_3 _was associated with effective anticancer therapy [16,17].

Previous studies documenting leukocyte infiltration in ventilator-induced lung injury (VILI) have focused on established injury [18]. However, the activity of leukocytes can be independent of the number of infiltrated cells, and the behavior of such leukocytes may depend on the stage of development of the injury and on the approach to ventilation. Although protective ventilation appears to affect the regional distribution of inflammation [19], it is not known whether ventilation strategy influences regional inflammatory cellular metabolic activity (for example, assessed by *k*_3_). PET-based methodology can be used to quantify regional lung ^18^F-FDG kinetics during ALI [20] and other pulmonary inflammatory conditions. We have previously shown in a model of two-hit ALI (VILI + endotoxemia) that both regional aeration and perfusion are associated with regional ^18^F-FDG uptake [8]. In the current study, we combined ^18^F-FDG-PET measurements of neutrophilic inflammation with PET assessments of perfusion and aeration in a sheep model to determine the impact of ventilation strategy on regional cellular metabolic activity during endotoxemia and to establish the association of this activity with regional changes in aeration and perfusion. We hypothesized that, during early endotoxemic lung injury, protective mechanical ventilation would reduce regional cellular metabolic activity (*k*_3_) and reduce the magnitude and heterogeneity of ^18^F-FDG uptake.

## Materials and methods

### Experimental preparation

The experimental protocols were approved by the Committee on Animal Care of the Massachusetts General Hospital (Boston, MA, USA). Twelve sheep (mean weight = 22.3 ± 5.9 kg) were fasted overnight and premedicated with intramuscular ketamine (4 mg/kg) and midazolam (2 mg/kg). After intravenous induction of anesthesia with ketamine (4 mg/kg), an endotracheal tube was inserted, in addition to a percutaneous femoral artery catheter (for arterial blood sampling and blood pressure monitoring) and a pulmonary artery catheter (right internal jugular vein) [10]. General anesthesia was maintained with a continuous infusion of propofol and fentanyl [8], and muscle relaxation was maintained with pancuronium (0.1 mg/kg).

### Mechanical ventilation

Baseline ventilation during experimental preparation (approximately 1.5 h) consisted of 8 ml/kg tidal volume (*V*_T_), 5 cmH_2_O positive end-expiratory pressure (PEEP) and 20 breaths/min respiratory rate. Prior to endotoxin administration (see the "Experimental protocol" section below), animals were allocated sequentially to protective ventilation (low *V*_T _and high PEEP) or injurious ventilation (high *V*_T _and low PEEP), which was continued for 2 h. Protective ventilation comprised 8 ml/kg *V*_T _and titration of PEEP such that the plateau pressure (*P*_plat_) was 30 cmH_2_O [3]. These settings were based on a clinically relevant strategy aimed at maximizing alveolar recruitment while limiting hyperinflation [3]. Injurious ventilation consisted of zero PEEP (0 cmH_2_O) and titrated *V*_T_, such that *P*_plat _was 30 cmH_2_O. This strategy was aimed at producing both cyclic recruitment-derecruitment and volutrauma while limiting hyperinflation by applying the same *P*_plat _levels used in the protective strategy. Volume-controlled ventilation was used in all cases.

A recruitment maneuver (continuous positive airway pressure of 40 cmH_2_O during 40 s [21]) was performed at the beginning of the experiment, before adjusting PEEP or *V*_T_. Inspired fraction of oxygen (FiO_2_) was titrated to a target arterial oxygen saturation greater than 88%, a 1:2 inspiratory-to-expiratory time ratio and an 18 breaths/min respiratory rate and adjusted to maintain arterial carbon dioxide tension at 32 to 45 mmHg.

#### Positron emission tomography imaging protocol and processing

The imaging methods and analysis we used have been described in detail previously [10,22-24]. PET images consisted of 15 axial slices (slice thickness = 6.5 mm), corresponding to approximately 70% of the lung [24]. Three different modalities of PET scans were performed: (1) transmission scans to correct for attenuation in emission scans and to calculate *f*_gas _from regional tissue density, which was used to categorize the pulmonary parenchyma as nonaerated (*f*_gas _< 0.1), poorly aerated (0.1 ≤ *f*_gas _< 0.5), normally aerated (0.5 ≤ *f*_gas _< 0.85) and hyperinflated (*f*_gas _≥ 0.85) [25]; (2) ^13^N[nitrogen] (^13^NN) emission scans using a bolus injection of ^13^NN-saline to measure regional pulmonary perfusion and shunt fraction [23,26]; and (3) ^18^F-FDG emission scans to quantify regional metabolic activity using ^18^F-FDG kinetics. Doses of 5 to 10 mCi of ^18^F-FDG were infused for 60 s at a constant rate through the central venous catheter. Acquisition time of the dynamic PET scans was 75 min starting simultaneously with the beginning of ^18^F-FDG infusion.

The lung field was delineated using perfusion and gas fraction images [11,27,28]. The whole field was divided for analysis into three horizontal adjacent regions of interest (ROIs) of equal vertical height (nondependent, middle and dependent).

### Modeling of ^18^F-fluorodeoxyglucose kinetics

**Figure 1:**
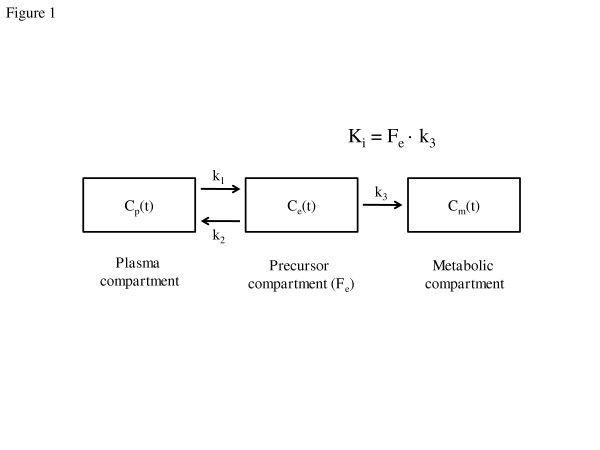
**Sokoloff model for ^18^F-fluorodeoxyglucose tracer kinetics **[13]. The three compartments of the model describe the activity concentration of ^18^F-fluorodeoxyglucose (^18^F-FDG) in plasma (*C*_p_(*t*)), the region of interest (ROI) concentration of extravascular ^18^F-FDG serving as a substrate pool for hexokinase (precursor compartment, *C*_e_(*t*)) and the ROI concentration of phosphorylated ^18^F-FDG (*C*_m_(*t*)). The arrows indicate the tracer exchanges in the dynamic model and the corresponding parameters. The rate constants *k*_1 _and *k*_2 _account for forward and backward transport of ^18^F-FDG between blood and tissue. *k*_3 _is the rate of ^18^F-FDG phosphorylation, reflecting hexokinase activity. The constant *F*_e _represents the distribution volume of ^18^F-FDG (that is, the substrate pool for hexokinase) as a fraction of lung tissue volume.

Inside cells, ^18^F-FDG is phosphorylated by hexokinase to ^18^F-FDG-6-phosphate, which accumulates in proportion to cellular metabolic rate. ^18^F-FDG net uptake rate was calculated by fitting the ^18^F-FDG kinetics with the Sokoloff three-compartment model for three isogravitational ROIs defined along the vertical axis: dependent, middle and nondependent [13]. This model is composed of one blood compartment and two tissue compartments, with the latter two representing a precursor and a metabolic compartment (Figure 1). The transfer rate constant *k*_3 _in this model characterizes ^18^F-FDG phosphorylation to ^18^F-FDG-6-phosphate (metabolic compartment), which is proportional to hexokinase activity and has been associated with cellular metabolic activity [16,29]. The ^18^F-FDG net uptake rate is computed as *K*_i _= *F*_e _× *k*_3_, where *F*_e _is the distribution volume of ^18^F-FDG as a fraction of lung volume [13,20,30].

To account for potential effects of lung inflation and blood volume on regional *K*_i_, we also standardized *K*_i _by lung tissue fraction, thus computing a specific *K*_i _as follows: *K*_is _= *K*_i_/*f*_tissue_, where *f*_tissue _= (1 − *f*_gas _− *f*_blood_) and *f*_blood _is the fractional volume of the blood compartment obtained from the Sokoloff model. *K*_is _is proportional to ^18^F-FDG uptake per gram of lung tissue. The Patlak two-compartment model [31] was used to compute ^18^F-FDG net uptake rate at the voxel level (*K*_iP_) to calculate the spatial heterogeneity of ^18^F-FDG uptake using the standard deviation (SD(*K*_iP_)) and to construct parametric images [20].

### Experimental protocol

Each sheep was placed supine in the PET scanner with the caudal end of the field of view just superior to the dome of the diaphragm. Physiological data and transmission and ^13^NN emission scans were acquired both at the start and after 2 h of mechanical ventilation, and the ^18^F-FDG scan was performed at the end of the study. After the initial set of scans, all sheep received a continuous infusion of endotoxin (*Escherichia coli *O55:B5, 10 ng/kg/min intravenously; List Biological Laboratories Inc, Campbell, CA, USA).

### Histological analysis

Lungs were excised at the end of the experiment and fixed with Trump's fixative (BBC Biochemical, Mt Vernon, WA, USA) at a pressure of 25 cmH_2_O. Blocks of lung tissue were sampled from ventral and dorsal regions and embedded in paraffin. Sections of 5-μm thickness were cut, mounted and stained with hematoxylin and eosin for light microscopy. Lung neutrophil counts and semiquantitative ALI scores [32] were assessed in 40 randomly selected high-power (×400 magnification) fields per animal (10 per region, 2 regions per lung) by two investigators (NP and MT) who were blinded to the group assignment. This procedure included two steps. First, a JPEG picture was obtained for each field and analyzed using dedicated software (Image-Pro Plus version 6.0; MediaCybernetics, Rockville, MD, USA). Each neutrophil was tallied and marked independently by investigators, and an overlay image was created. Second, for each single field, the values obtained were compared and, in cases of discrepancies in neutrophil counts between investigators, the histologic images were compared and discrepant neutrophil marks were discarded. This approach might have led to a slight underestimation of neutrophil counts but ensured better specificity. A final neutrophil count was obtained and expressed as neutrophils/field/unit of tissue, whereupon normalization for the tissue fraction of each field was performed to account for differences in regional lung inflation. Independent investigators had good agreement as to neutrophil counts in the injurious ventilation group (Lin's concordance correlation coefficient = 0.95) and the assessment of lung injury scores (κ = 0.65).

#### Statistical analysis

Data are expressed as mean ± SD if normally distributed or, if not, as median [25% to 75% interquartile range]. We compared physiological values (before and after mechanical ventilation and between ventilation groups) and PET-acquired data (between ventilation groups and isogravitational ROIs) using two-way analysis of variance for repeated measures when data or their log transformation displayed a normal distribution. Data that displayed a nonparametric distribution were compared using the Friedman rank-sum test.

## Results

### Baseline physiological variables

Protective ventilation yielded lower cardiac output (*P *< 0.05) and higher pulmonary vascular resistance (*P *< 0.001) after 2 h of mechanical ventilation and endotoxemia, despite similar baseline values (Table 1). Blood neutrophil counts decreased significantly in both groups following endotoxin administration (*P *< 0.05).

**Table 1:** Physiological variables and peripheral neutrophil counts at baseline and 2 hours after mechanical ventilation and endotoxemia.^a^

	Injurious ventilation (*n *= 6)		Protective ventilation (*n *= 6)	
	
Parameter	Baseline	After MV + ETX	Baseline	After MV + ETX
*V*_T_, ml/kg	18.6 ± 3.9^b^	14.3 ± 4.4^c^	8.1 ± 0.2	8.0 ± 0.2
RR, breaths/min	19 ± 2^b^	21 ± 3^c^	25 ± 2	26 ± 2
PEEP, cmH_2_O	0^b^	0^*a*^	17 ± 3	17 ± 2
Mean airway pressure, cmH_2_O	10 ± 2^b^	10 ± 1^b^	20 ± 3	21 ± 2
FiO_2_	0.30 (0.30 to 0.35)	0.35 (0.30 to 0.62)	0.30 (0.30 to 0.40)	0.30 (0.30 to 0.42)
PaO_2_/FiO_2_, torr^g^	255 ± 74^c^	162 ± 67^d^	351 ± 117	261 ± 112
PaCO_2_, torr	33 (30 to 40)	43 (41 to 46)	39 (36 to 42)	39 (33 to 43)
*Q*_s_/*Q*_t_	0.24 (0.03 to 0.49)	0.60 (0.37 to 0.75)	0.15 (0.07 to 0.36)	0.37 (0.21 to 0.48)
*C*_rs_, ml/cmH_2_O	15.4 ± 6.8	11.4 ± 3.5	12.8 ± 2.7	12.2 ± 1.8
Heart rate, bpm	188 ± 30^c^	150 ± 41^f^	120 ± 26	137 ± 34
Cardiac output, L/min	4.3 ± 1.1	4.4 ± 0.7^d^	3.3 ± 0.7	3.1 ± 0.5
MAP, mmHg	92 ± 12	78 ± 13	87 ± 13	81 ± 13
MPAP, mmHg	16 ± 9	21 ± 5^d^	22 ± 6	32 ± 6^e^
PVR, dyn/s/cm^5^	228 ± 97	313 ± 109^b^	372 ± 108	579 ± 118^f^
Blood neutrophil count (10^3^/μl)^g^	3.09 (1.32 to 3.96)	0.23 (0.14 to 0.25)^e^	1.80 (1.05 to 3.90)	0.20 (0.02 to 2.17)

^a^MV, mechanical ventilation; ETX, endotoxin; *V*_T_, tidal volume; RR, respiratory rate; PEEP, positive end-expiratory pressure; FiO_2_, fraction of inspired oxygen; PaO_2_/FiO_2_, ratio of partial pressure of oxygen in arterial blood to fraction of inspired oxygen; PaCO_2_, arterial carbon dioxide tension; *Q*_s_/*Q*_t_, venous admission calculated with the Berggren equation; *C*_rs_, respiratory system compliance; MAP, mean arterial pressure; MPAP, mean pulmonary arterial pressure; PVR, pulmonary vascular resistance. Comparisons between both groups at the same time point: ^b^*P *< 0.001, ^c^*P *< 0.01 and ^d^*P *< 0.05. Comparison between baseline and after mechanical ventilation and endotoxemia within each group: ^e^*P *< 0.01 and ^f^*P *< 0.05. ^g^*P *< 0.05 for global effect of time. *P *values are derived from two-way analysis of variance with Bonferroni adjustments on *post hoc *tests.

### Oxygenation, regional aeration and perfusion before endotoxin administration

**Figure 2:**
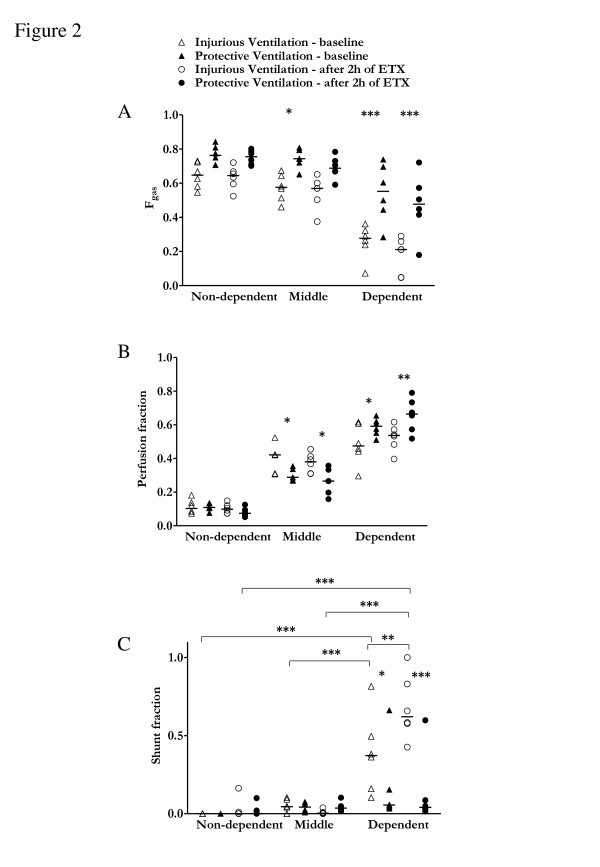
**Regional gas, perfusion and shunt fractions**. Gas fraction (*f*_gas_) **(A)**, perfusion fraction **(B) **and shunt fraction **(C) **for isogravitational (dependent, middle and nondependent) regions of interest of injurious (open symbols) and protective ventilation (filled symbols) groups at baseline (triangles) and after (circles) 2 h of mechanical ventilation and endotoxemia (ETX). *f*_gas _and perfusion fraction were stable over time in both groups. In contrast, shunt fraction dramatically increased over time in dependent regions of the injurious ventilation group, but not in those of the protective ventilation group. Horizontal lines represent median values. *P < 0.05, **P < 0.01 and ***P < 0.001. *P *values are derived from two-way analysis of variance with repeated measurements and Bonferroni adjustments for multiple comparisons.

Before endotoxin administration, protective ventilation was associated with a higher overall lung gas fraction (*f*_gas _= 0.71 ± 0.12 vs. 0.48 ± 0.08; *P *= 0.004) and a better ratio of partial pressure of oxygen in arterial blood (PaO_2_) to FiO_2 _(PaO_2_/FiO_2 _= 351 ± 117 mmHg vs. 255 ± 74 mmHg; *P *< 0.01) compared with injurious ventilation, despite no difference in venous admixture (0.15 [0.07 to 0.36] vs. 0.24 [0.03 to 0.49]; *P *= 0.89) (Table 1). Protective ventilation resulted in higher degrees of regional *f*_gas _in the middle and dependent lung regions (Figure 2A) compared with injurious ventilation. The amount of hyperinflated voxels was lower than 1% in both the protective and injurious ventilation groups.

Regional perfusion fraction with protective (vs. injurious) ventilation was lower in middle regions and higher in dependent regions (Figure 2B). There was a significant vertical dependence of perfusion in both groups at baseline (*P *< 0.01). Regional shunt also varied with isogravitational region (*P *< 0.001), but not with ventilation strategy, and was lower in dependent regions with protective vs. injurious ventilation (Figure 2C).

### Oxygenation, regional aeration and perfusion after endotoxin administration

Following 2 h of endotoxin administration and mechanical ventilation, oxygenation decreased significantly in both groups (*P *< 0.05). Global and regional (Figure 2A) lung aeration for both groups remained stable. Perfusion distribution to each isogravitational lung region was also maintained (Figure 2B). However, with injurious ventilation, there was a significant increase in shunt fraction in the dependent lung regions (*P *< 0.01), which did not occur with protective ventilation (Figure 2C).

Compared to injurious ventilation, protective ventilation after 2 h of endotoxemia was associated with better oxygenation (PaO_2_/FiO_2 _= 261 ± 112 torr vs. 162 ± 47 torr; *P *= 0.05) and a trend toward lower venous admixture (0.37 [0.21 to 0.48] vs. 0.60 [0.37 to 0.75]; *P *= 0.06) (Table 1). Protective ventilation after endotoxin administration was associated with a lower perfusion fraction in middle regions (0.26 ± 0.08 vs. 0.37 ± 0.06; *P *< 0.05) but a higher perfusion fraction in dependent regions (0.66 ± 0.10 vs. 0.52 ± 0.08; *P *< 0.05) (Figure 2B). Regional shunt varied with isogravitational region (*P *< 0.001) and ventilation strategy (*P *< 0.01), with significant interaction (*P *< 0.001), and was reduced in dependent regions in the protective ventilation group (Figure 2C).

### ^18^F-fluorodeoxyglucose uptake magnitude and topographical distribution

**Figure 3:**
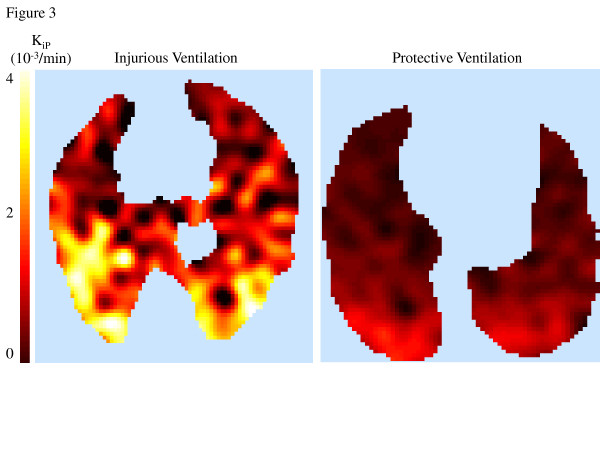
**Single-slice images of ^18^F-fluorodeoxyglucose uptake rate**. Single-slice images of ^18^F-fluorodeoxyglucose (^18^F-FDG) uptake rate (*K*_iP_, computed voxel-by-voxel using the Patlak method [31]) in one sheep from the protective ventilation group (right panel) and in one from the injurious ventilation group (left panel). Left side in each image corresponds to the left side in the animal. ^18^F-FDG uptake was lower and more homogeneous in the protective ventilation experiment.

**Figure 4:**
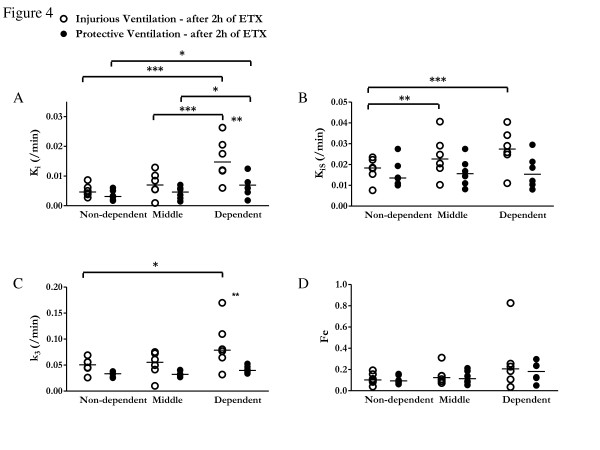
**^18^F-fluorodeoxyglucose kinetics parameters**. ^18^F-fluorodeoxyglucose (^18^F-FDG) kinetics parameters for isogravitational (dependent, middle and nondependent) regions of interest of injurious (open circles) and protective (closed circles) ventilation groups. **(A) **^18^F-FDG net uptake rate (*K*_i_). **(B) **^18^F-FDG net uptake rate normalized for tissue density and blood fraction (*K*_is _= *K*_i_/(1 − *f*_gas _− *f*_blood_)); C) Transfer rate constant *k*_3 _(characterizes ^18^F-FDG phosphorylation to ^18^F-FDG-6-phosphate and reflects hexokinase activity); and D) Precursor compartment for ^18^F-FDG phosphorylation (*F*_e_). Note the significant regional heterogeneity in the distribution of K_is _and k_3 _in the injurious as compared to the protective ventilation group. Horizontal lines represent median values. *P < 0.05, **P < 0.01 and *** *P *< 0.001. *P *values are derived from two-way analysis of variance with repeated measurements, with Bonferroni adjustments for multiple comparisons.

After endotoxin and 2 h of mechanical ventilation, global *K*_i_, computed using the Sokoloff model, tended to be lower in the protective ventilation group (0.0056 ± 0.0029/min vs. 0.0102 ± 0.0053/min; *P *= 0.09) (Figure 3). At the regional level, there was a significant effect of isogravitational region (*P *< 0.001) and a trend toward a significant effect of ventilation strategy on *K*_i _(*P *= 0.059) with significant interaction (isogravitational region × ventilation strategy; *P *= 0.005) (Figure 4A). In the dependent regions, *K*_i _was lower with protective ventilation than with injurious ventilation (0.0075 ± 0.0043/min vs. 0.0157 ± 0.0072/min; *P *< 0.01) (Figure 4A). When differences in lung inflation and tissue volume were accounted for (*K*_iS_) (Figure 4B), the significant interaction between ventilation strategy and isogravitational region on *K*_iS _was maintained (*P *= 0.004), as was the independent effect of isogravitational region (*P *= 0.001).

In order to understand the factors contributing to the changes in regional *K*_i_, we studied its components: the cellular metabolic activity (*k*_3_) and the distribution volume of ^18^F-FDG as a fraction of lung volume (*F*_e_, where *K*_i _= *k*_3 _× *F*_e_). *k*_3 _was significantly lower with protective ventilation than with injurious ventilation (*P *= 0.002) and was highest in dependent regions of injurious ventilation (Figure 4C). In contrast, *F*_e _varied significantly by isogravitational region (*P *= 0.022) (Figure 4D).

**Figure 5:**
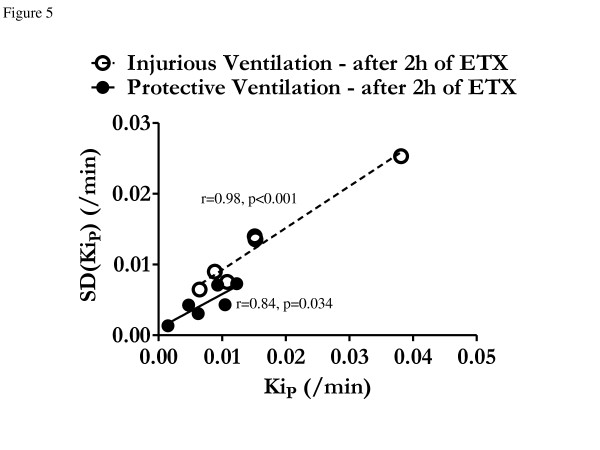
**Correlation between mean ^18^F-fluorodeoxyglucose net uptake rate at the voxel level and ^18^F-fluorodeoxyglucose uptake using the standard deviation**. Linear regression between mean (*K*_iP_) and standard deviation (SD(*K*_iP_)) of voxel-level ^18^F-fluorodeoxyglucose (^18^F-FDG) net uptake rate computed using the Patlak method [31] for protective (filled circles) and injurious (open circles) ventilation groups. There was a significant correlation between mean *K*_iP _and SD(*K*_iP_) for both the protective ventilation group (continuous line; *y *= 0.48*x *+ 0.001, *r *= 0.84, *P *= 0.034) and the injurious ventilation group (dashed line; *y *= 0.59*x *+ 0.003, *r *= 0.98, *P *< 0.001). Note the offset between the two regression lines showing that, for equivalent *K*_iP _values, protective ventilation led to lower SD(*K*_iP_) than injurious ventilation.

Protective (vs. injurious) ventilation was associated with a threefold reduction in the spatial heterogeneity of pulmonary ^18^F-FDG uptake (indicated by SD(*K*_iP_); 0.0045 ± 0.0023/min vs. 0.0126 ± 0.0069/min; *P *< 0.01), reflecting a more homogeneous distribution of metabolic activation throughout the lung. The heterogeneity of ^18^F-FDG uptake distribution SD(*K*_iP_) correlated with *K*_iP _in both groups, with similar slopes (Figure 5); however, the protective ventilation group exhibited a lower *y*-intercept (0.0009 ± 0.0013/min vs. 0.0033 ± 0.0012/min; *P *< 0.01), such that for equivalent mean *K*_i _values, the heterogeneity of *K*_i _was lower with protective ventilation than with injurious ventilation.

### Regional lung histology and ^18^F-fluorodeoxyglucose uptake values

**Figure 6:**
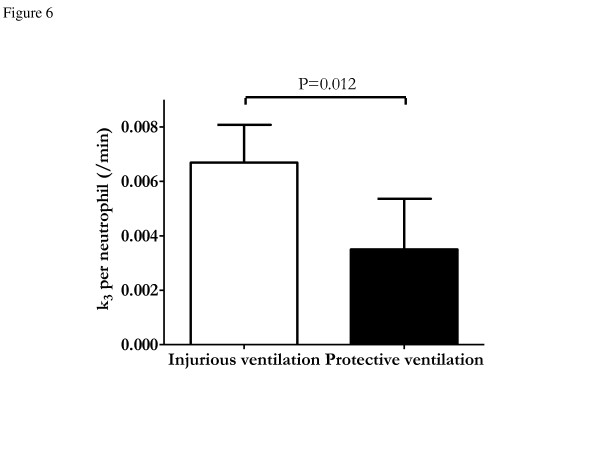
**Metabolic activity per neutrophil in dependent lung regions**. Neutrophils of the injurious ventilation group exhibited higher *k*_3 _values than those of the protective ventilation group (*P *= 0.012), reflecting higher hexokinase activity.

Endotoxin infusion was associated with marked systemic neutropenia in both ventilation groups (Table 1). Median ALI scores were less than 2 on a scale of 12 in the dorsal and ventral regions of both groups, without a statistically significant difference (Table 2). No significant difference was observed in global lung neutrophil counts between the protective and injurious ventilation groups (53.2 ± 23.5/field/unit of tissue vs. 43.2 ± 11.6/field/unit of tissue; *P *= 0.24). At the regional level, there was no difference between groups (*P *= 0.33), but there was a significant effect of lung region on neutrophil count (*P *< 0.01) with no interaction (*P *= 0.87). In dependent regions, lung neutrophils of the injurious ventilation group exhibited higher *k*_3 _values than those of the protective ventilation group (Figure 6). There was a significant correlation between *F*_e _and regional neutrophil counts found in histological analysis (*r *= 0.43; *P *= 0.046).

**Table 2:** Lung histological analysis.^a^

	Injurious ventilation (*n *= 6)		Protective ventilation (*n *= 6)		*P*
		
Lung region	Ventral	Dorsal	Ventral	Dorsal	
Alveolar edema (*n *= 3)	1 [0 to 1]	1 [1 to 1]	0 [0 to 0]	0 [0 to 0]	0.32
Septal edema (*n *= 3)	0 [0 to 0]	0 [0 to 0]	1 [0 to 1]	1 [0 to 2]	0.16
Alveolar hemorrhage (*n *= 3)	0 [0 to 1]	1 [1 to 1]	0 [0 to 0]	0 [0 to 0]	0.16
Septal congestion (*n *= 3)	0 [0 to 0]	0 [0 to 0]	1 [0 to 1]	1 [0 to 1]	0.99
Total score (*n *= 12)	1 [0 to 2]	1 [1 to 2]	1 [0 to 2]	1 [0 to 3]	0.16

^a^*P *values are derived from a Friedman rank-sum test. Values are medians [25% to 75% percentiles].

## Discussion

Our main findings are that after only 2 h of the combined effects of endotoxemia and mechanical ventilation, a protective ventilation strategy that maximized alveolar recruitment and limited lung distension led to the following results: (1) decreased rate of phosphorylation of ^18^F-FDG (*k*_3_), which reflects metabolic cellular activity; (2) reduced heterogeneity of ^18^F-FDG uptake rate throughout the lung; (3) decreased magnitude of ^18^F-FDG uptake in the whole lung, predominantly by reducing ^18^F-FDG uptake in the poorly aerated, dependent lung regions, despite greater perfusion, and thus more endotoxin exposure in these areas in the protective than in the injurious ventilation strategy; and (4) no reduction in the number of lung-infiltrating neutrophils. These findings suggest an effect of the protective ventilation strategy with low *V*_T _in modulating the intensity and topographical distribution of regional pulmonary neutrophilic inflammation during mechanical ventilation following endotoxemia. The intensity of inflammation was best reflected in reduction of inflammatory cell metabolic activity (that is, *k*_3_), even when regional neutrophil numbers were similar.

### Animal model

We used a well-established model of endotoxemia because of its reproducibility, its ability to induce pulmonary vascular neutrophil sequestration within the first hour [33] and thus its relevance to study neutrophil activation [34]. In previous studies, the current endotoxin dose allowed for survival of awake animals for 24 h [35,36]. Thus, in terms of lung injury, the initial 2 h of endotoxin exposure represent early events, as attested by very low lung injury scores obtained by histological analysis. The scatter of PaO_2_/FiO_2 _ratio values obtained after 2 h of mechanical ventilation and endotoxin illustrates the intersubject variability of lung function impairment, even after standardized lung injury. Nevertheless, the protective ventilation strategy produced significantly higher PaO_2_/FiO_2 _levels than the injurious ventilation strategy. Our regional PET measurements confirmed that the model also yields a broad range of regional lung expansion with minimal hyperinflation (no regional *f*_gas _value above 0.9) and appropriately reflects the lung recruitment and reduction in shunting in dependent lung regions produced by protective ventilation. In addition, the approach to protective ventilation chosen in the current experiments closely reflects the management strategy used in one of the key randomized controlled clinical trials of acute respiratory distress syndrome (ARDS) [3], in which *V*_T _was fixed and the level of PEEP adjusted to a predetermined *P*_plat_, although important differences include the volume preset ventilation used in the current study as well as the normal initial lung compliance. In fact, we chose to set the *V*_T _at 8 ml/kg to limit alveolar hypoventilation related to the larger anatomic dead space in sheep than in humans (normal *V*_T _in spontaneously breathing sheep is approximately10 ml/kg) [37]. That *V*_T _setting provided a clear distinction from the injurious model, although it exceeded the current clinical recommendation for *V*_T _of 6 ml/kg of predicted body weight in ARDS patients [38,39] and even lower settings (4.2 ml/kg) for ARDS patients with high *P*_plat _[40]. Hemodynamics remained stable over time, which is consistent with the administration of mild doses of endotoxin. The lower cardiac output in the protective group was likely due to higher intrathoracic pressure associated with higher PEEP levels.

### Early changes in regional ^18^F-fluorodeoxyglucose uptake during acute lung injury

In the acutely injured lung, PET imaging of the glucose analogue ^18^F-FDG functions as a noninvasive *in vivo *measure of neutrophilic inflammation [7,10,12,41]. Recent human and animal studies of ALI and ARDS indicate that total ^18^F-FDG uptake may provide insight into disease mechanisms [8,10,11,42,43] and predict respiratory failure [44], as well as being useful in evaluation of therapy [45]; however, the impact of ventilation strategy (that is, protective vs. injurious) on intensity or topographic activity has not previously been reported. The current findings indicate, at least in the current model, that mechanical ventilation has an important effect in determining the regional distribution and degree of early neutrophilic inflammation. Protective ventilation resulted in more homogeneously distributed *K*_i _and lower *K*_i _values in dependent lung regions. Furthermore, we observed a trend toward reduction in pulmonary ^18^F-FDG uptake with protective ventilation, despite similar histological lung injury scores and equal end-inspiratory pressures. These results are compatible with the concept that ventilation strategy plays an early pathogenic role in determining the profile of inflammatory cell distribution before lung injury is established [46] and emphasize that the prevention of VILI should be a key aspect of patient management, even when mechanical ventilation period and the underlying level of injury are limited [47].

Our *in vivo *measurements substantiate prior speculation that heterogeneous inflammation may be an important element of the pathogenesis of ALI and ARDS [19,48]. However, there are conflicting data on the topography of early lung inflammation during ALI and ARDS. A previous study in a surfactant-depleted small animal model (saline lavage rat model) indicated that aerated, nondependent regions were those predominantly affected by mechanical ventilation without limitation of end-inspiratory pressure [49]. In that study, surfactant depletion promoted the derecruitment of dependent regions and thus likely led to overexpansion of nondependent regions substantially more than in the injurious ventilation group in the current study. This phenomenon was amplified by the application of larger *V*_T _in the rat model (25 ml/kg), resulting in a much larger nondependent strain [49]. Recently, researchers in a study in which a sheep model of endotoxemic ARDS and mechanical ventilation was used reported that inflammatory changes occurred predominantly in apical lung regions, but not in basal regions [50]. Such topographic differences with our injurious ventilation group might be related to the application of PEEP, which prevented the development of inflammation in dorsal regions, likely through the reduction of low lung volume mechanisms. Our results in an animal model of size comparable to the human and using clinically relevant *P*_plat _limits indicate that, in early endotoxemia during mechanical ventilation without PEEP, inflammation occurs in the dependent regions. This suggests that those regions may be targeted as key foci indicating inflammatory activity with the potential for PET imaging to quantify treatment response.

There is controversy regarding the relationship between regional ^18^F-FDG uptake and regional aeration and perfusion in heterogeneous lungs [51]. In a model of endotoxemic ALI and mechanical ventilation [8], we previously suggested that higher regional ^18^F-FDG uptake was associated with regional extremes in aeration (low and high) and high perfusion. This suggested an effect of both low-volume lung injury and hyperinflation on ^18^F-FDG uptake and presumably indicated a link between regional neutrophilic inflammation and regional exposure to endotoxins, inflammatory mediators and cells. In this context, the reduced ^18^F-FDG uptake in dependent regions of the protective ventilation group, despite an increase in perfusion of these regions, suggests that the effect of the protective ventilation strategy on reducing ^18^F-FDG uptake was predominantly related to increased regional aeration. This effect could have been partially due to the reduction in the shunt fraction of dependent regions, as hypoxemia promotes neutrophil influx into shunting regions through increased neutrophil-endothelium interaction [52].

### Cellular factors contributing to ^18^F-fluorodeoxyglucose uptake

Pulmonary ^18^F-FDG uptake is determined by both cell numbers and cellular metabolic activity [20,30,41]. Individual cell activation is associated with increased energy requirements, and enhanced glucose uptake (that is, cellular metabolic activity) reflects the energy involved in key functions (for example, migration, phagocytosis, degranulation, generation of toxic reactive oxygen intermediates and cytokine production) [53,54]. Quantification of cell activation independent of the inflammatory cell number may be particularly relevant during ALI, given the major role of neutrophil activation in the early stages of ALI [55]. Indeed, previous studies have suggested that lung injury is affected mainly by neutrophil activation rather than by their number [41,55,56] that the transfer rate (*k*_3 _in this analysis) obtained from the analysis of ^18^F-FDG kinetics associated with hexokinase activity [13,20] allows for such assessment [16].

Our results suggest that the effect of ventilation strategy in reducing ^18^F-FDG uptake during early endotoxemia was predominantly through its effect on cellular metabolic activity (*k*_3_), not on cell number (*F*_e_). Metabolic activity, *k*_3_, was the predominant contributing factor to the reduction in ^18^F-FDG uptake by protective (vs. injurious) ventilation and was significantly reduced in dependent lung regions in the protective ventilation group. Given that the cells taking up ^18^F-FDG are predominantly neutrophils in this model [7,8,12,30,41], the lower *k*_3 _value mainly reflects lower metabolic activity of lung-infiltrating neutrophils during protective ventilation with higher PEEP and lower *V*_T_.

This predominant role of *k*_3 _was confirmed by the finding of lower *K*_i _with protective vs. injurious ventilation for a similar range of regional neutrophil quantities in both groups, measured independently and using direct histological methods. Moreover, we found lower *k*_3 _values per neutrophil in dependent regions of the protective (vs. injurious) ventilation group (Figure 6). This novel finding leads us to speculate that a reduction in lung neutrophil activity could be a mechanism by which protective ventilation improves outcomes of ALI and ARDS patients [3,38]. This result also suggests that quantification of cell activation (*k*_3_) allows for characterization of differences in the type and severity of ALI, even when inflammatory cell numbers are similar. This observation is compatible with the reported relationship between *k*_3 _and severity of disease in cancer research, where cell activity was indicated as a marker of severity [14,16,17], and suggests that *k*_3 _could be a sensitive tool with which to monitor noninvasively early changes in lung inflammation in ALI and ARDS. The observed dissociation between cell numbers and metabolic activity suggested by the current study in an experimental model of ALI paves the way for the development of new methods for quantifying the effects of mechanical or pharmacological interventions in ALI and ARDS.

### Factors associated with modulation of lung inflammation by a protective ventilation strategy

Different factors could explain the reduction in neutrophil activation associated with protective ventilation. First, there are those related to regional lung mechanics. Given that the same *P*_plat _was used in all animals, group differences in ^18^F-FDG uptake should be due to different PEEP volume and *V*_T_. Because significant differences in ^18^F-FDG uptake were evident in the dependent poorly aerated lung regions, low-volume lung injury is a likely factor. Such injury is related to processes such as repetitive opening and closing of distal airways and alveoli [57,58], concentration of regional mechanical forces [59] and propagation of air in fluid-filled airways [60]. In fact, this low-volume effect associated with smaller PEEP levels would be magnified by the concomitant increase in *V*_T _[61]. Because dependent lung units with mechanical instability express higher cytokine levels than nondependent units [19], reduction in the number of those units by protective ventilation could decrease the activation of inflammatory cells and result in lower *K*_i _independently of the number of neutrophils.

The linear relationship between SD(*K*_ip_) and *K*_ip _for both groups is suggestive of a positive feedback mechanism in the generation of the spatial distribution of *K*_ip_. Points were tightly distributed around the regression line and showed an increase in *K*_ip _heterogeneity as the average inflammation level increased, with an approximately constant coefficient of variation. The coefficient of variation is related by a monotonic, increasing transformation to the standard deviation of a log-normal distribution [62]. This distribution describes multiplicative phenomena and is frequently encountered in lung physiology [63,64]. We speculate that a multiplicative factor in our study could have been produced by triggering of regional lung inflammation with parenchymal cell activation and release of inflammatory mediators. This would result in chemotaxis and additional regional cellular activation, which would amplify the inflammatory process [5,64]. In contrast, less inflamed lung regions would not demonstrate such amplification. These changes would result in an increase in the mean and SD *K*_ip _values. Our results suggest that such an increase occurs according to a well-defined quantitative relationship.

### Methodological considerations and limitations

First, we used two strategies of ventilation (low *V*_T_/high PEEP vs. high *V*_T_/low PEEP) designed to produce clearly different degrees of lung inflation magnitude and heterogeneity in order to test the effect of mechanical ventilation on regional cellular metabolic activity. Thus, our results cannot be directly extrapolated to the clinical context. In fact, whereas the maximal recruitment strategy produced a trend towards lower mortality in patients with severe ARDS, it led to the opposite trend in patients with predominantly mild ARDS (PaO_2_/FiO_2 _>181 mmHg) [3]. Accordingly, further studies will be needed to exactly determine settings consistent with "protective ventilation" for the individual patient. Second, the observed relation between histological neutrophil counts and the distribution volume of ^18^F-FDG (*F*_e_) was weak, suggesting that other cell types contributed to ^18^F-FDG uptake, as previously shown [65]. The cellular mechanisms associated with increased ^18^F-FDG uptake, specifically changes in *k*_3_, were not investigated in this study and could involve metabolic changes associated with the polarization and migrational status of lung neutrophils [54], Toll-like receptor 4-dependent mechanisms [66,67] and the regional production of neutrophil chemoattractant cytokines [68].

## Conclusion

Our present study shows that, at early stages of endotoxemic ALI, mechanical ventilation strategy strongly influences the intensity and distribution of neutrophil inflammation in the lungs. Protective ventilation (increased alveolar recruitment, low *V*_T_) improved gas exchange and reduced inflammatory cell activity, especially in dependent lung regions. The main cause of reduced inflammatory cell activity was lowered neutrophilic metabolic activity (phosphorylation rate), not changes in regional cell counts. This suggests that mechanical ventilation may modulate regional neutrophilic inflammation in early stages of endotoxemic ALI.

## Key messages

• At early stages of endotoxemic ALI, mechanical ventilation strategy influences the intensity and distribution of neutrophil inflammation in the lungs.

• Protective ventilation improved gas exchange and reduced inflammatory cell activity in dependent lung regions.

• The main cause of reduced inflammatory cell activity was lowered neutrophilic metabolic activity.

## Abbreviations

ALI: Acute lung injury; ^18^F-FDG: ^18^F-fluorodeoxyglucose; *f*_gas_: Gas fraction; PET: Positron emission tomography; ROI: Region of interest; VILI: Ventilator-induced lung injury.

## Competing interests

The authors declare that they have no competing interests.

## Authors' contributions

NP, EC, TWe, TWi, BK and MVM designed the study. NP, EC, TWe, MT, TWi, GM, RH, JV and MVM performed the experiments and analyzed the results. NP, EC, TWe, GM, MT, TWi, RH, JV, BK and MVM wrote and corrected the manuscript. All authors read and approved the final manuscript.
